# Supplementary material for: Dynamics of Endothelial Engagement and Filopodia Formation in Complex 3D Microscaffolds
Source: Int J Mol Sci. 2022 Feb 22;23(5):2415. doi: 10.3390/ijms23052415 (PMC8910162; doi:10.3390/ijms23052415)
Supplement: Supplementary file 1 [file ijms-23-02415-s001.zip › Supplementary Materials.pdf]

Supplementary Materials

Dynamics of Endothelial Engagement and Filopodia Formation in Complex 3D Microscaffolds

Typical movies of HUVECs Lifeact-GFP on microstructures are shown (all movies are accessible in the raw data, see Data Availability Statement). The elapsed time (in minutes) is indicated on top, left, of the movies. **Movie S1a,b**: HUVEC dynamics on a closed (Movie S1a) *l7L14-closed* microstructure and on an open (Movie S1b) *l7L14-open* microstructure, $\Delta t = 1$ min, *z* maximal projection of the bottom planes. Cells formed a confluent monolayer on top of the microstructure. **Movies S2–S12:** HUVEC dynamics in open *l7L14-open* microstructures, $\Delta t = 20$ s, maximal projection of the bottom planes. Pairs of movies refer to conditions before (first movie, beginning 20 min before drug addition) and immediately after drug addition (second movie of each pair). **Movie S2:** Spontaneous transition from an adherent (mesenchymal) state to a bleb (amoeboid) state. **Movie S3:** Spontaneous transition from a bleb (amoeboid) state to an adherent (mesenchymal) state. **Movies S4 and S5:** Before and after addition of ML-7 10 μM, **Movies S6 and S7**: Before and after addition of Y-27632 10 μM, **Movie S8:** Example of spontaneous membrane ruffling at the base of filopodia, giving birth to dactylopodia-like protrusions, **Movies S9 and S10:** Before and after addition of PF-573228 10 μM, **Movies S11 and S12:** Before and after addition of sunitinib 300 nM. Scale bars, 10 μm. **Movies S13 and S14**: A total of 5 h and 18 h after addition of sunitinib 300 nM (independent experiments).
